# Tracheostomy for COVID-19: evolving best practice

**DOI:** 10.1186/s13054-021-03674-7

**Published:** 2021-08-31

**Authors:** Thomas Williams, Brendan A. McGrath

**Affiliations:** 1 Academic Foundation Trainee, University Hospitals of Morecambe Bay NHS Foundation Trust, Lancaster, UK; 2 Acute Intensive Care Unit, Wythenshawe Hospital, Manchester University NHS Foundation Trust, Manchester, UK; 3 Manchester Academic Critical Care, Division of Infection, Immunity and Respiratory Medicine, School of Biological Sciences, Faculty of Biology, Medicine and Health, The University of Manchester, Manchester Academic Health Science Centre, Manchester, UK

## Abstract

This article is one of ten reviews selected from the Annual Update in Intensive Care and Emergency Medicine 2021. Other selected articles can be found online at https://www.biomedcentral.com/collections/annualupdate2021. Further information about the Annual Update in Intensive Care and Emergency Medicine is available from https://link.springer.com/bookseries/8901.

The global pandemic caused by the novel severe acute respiratory syndrome coronavirus 2 (SARS-CoV-2) has had a dramatic impact upon all areas of healthcare, and this is no more evident than in critical care. Management of the critically ill evolved over time, with variability in admission criteria and the use of invasive ventilation reported from around the world and within individual countries [1]. However, the majority of patients admitted to intensive care units (ICUs) required advanced respiratory support [1], often for longer periods than expected when compared with historical viral pneumonias [2]. Tracheostomy is an entrenched element of modern critical care, with the dominant indication established as facilitating long-term ventilation and ‘weaning’ from respiratory support. Additional indications include actual or threatened upper airway obstruction, facilitating pulmonary clearance and to offer a degree of ‘protection’ against pulmonary aspiration. Prior to this pandemic, tracheostomy could be anticipated in 8–13% of patients receiving advanced respiratory support in modern ICUs [3]; usually temporary, but often *in situ* for several weeks (a median of 28 days in one recent UK-wide study) [4]. Reported rates of tracheostomies utilized during the coronavirus pandemic vary significantly from 16% to 61% [5, 6], but are certainly significantly higher than pre-pandemic rates.

As with many aspects of management, our understanding of how best to employ tracheostomy during the pandemic has evolved. There are many potential benefits of tracheostomy for the patient and for stressed healthcare systems, which have led some institutions to employ tracheostomy relatively early in the patient’s ICU stay, but detailed outcome data from large case series are not available. Tracheostomy insertion and subsequent management also requires trained, equipped and supported staff to minimize the potential for complications and patient safety incidents [7]. It is essential that we understand which patients with coronavirus disease 2019 (COVID-19) may benefit from tracheostomy, along with when and how it should be employed. Importantly, in non-COVID-19 patients, only around 20% of tracheostomy patients survive beyond ICU discharge to 1 year [8], repeatedly raising questions about patient selection, which are relevant as hospitals around the world struggle to manage large volumes of critically ill patients. These problems are compounded in the pandemic with patients frequently managed in makeshift or unfamiliar settings, often by non-CU trained medical, nursing and allied healthcare professional staff.

In this state-of-the-art review, we consider these important issues affecting around one-fifth of critically ill patients presenting to our ICUs with severe respiratory failure resulting from COVID-19.

## Why perform a tracheostomy?

Tracheostomy can benefit patients who require prolonged ventilation: enabling sedation to be reduced or stopped; enabling the removal of the trans-laryngeal tube to facilitate laryngeal rehabilitation; and offering an interface for variable invasive ventilator support without having to resort to re-sedation and tracheal re-intubation [9]. Considering that patients with COVID-19 typically have longer periods of ventilation than patients with other viral pneumonias [2], it is not surprising that studies have demonstrated that tracheostomy for COVID-19 disease may confer a survival benefit [5], aid weaning from ventilatory support [6], and may ease the burden upon critical care resources [10]. Recent UK data highlighted that non-COVID-19 tracheostomy patients typically spend a median of 50 days in hospital, 28 days with a tracheostomy *in situ*, and 23 days within the ICU [4]. It is easy to appreciate how critical care resources may become overwhelmed following a surge in demand. When resources become stretched, decisions regarding resource allocation become more challenging, and difficult judgments balancing tracheostomy, prolonged ventilation, rehabilitation and the potential of providing a real benefit for long-term quality of life need to be made.

What makes a patient with COVID-19 different when considering tracheostomy? With the high transmissibility and risk of serious illness, the potential risks to healthcare staff need to be considered in addition to the potential benefits to the patient. One argument surrounds the challenges of primary extubation, with higher rates of reintubation reported in patients with COVID-19 [11]. Prolonged periods of tracheal intubation associated with the use of neuromuscular blocking agents [2] and the routine use of systemic corticosteroids [12] contribute to respiratory muscle deconditioning [13], which can make going straight from an endotracheal tube to self-supported breathing challenging. Urgent re-intubation of a critically hypoxic patient has clear risks for the patient, but it is also important to consider the risks to attending staff. Non-invasive ventilation, face-mask continuous positive airway pressure, or high-flow nasal oxygen pose potential risks to healthcare staff through infectious aerosol generation [14], compounded by the risks associated with re-intubation [15].

SARS-CoV-2 itself may contribute to the increased rates of laryngeal edema and pathology reported in patients with COVID-19 [16, 17]. It is difficult to distinguish whether laryngeal pathology is a consequence of coronavirus infection, a sequel to the associated prolonged tracheal intubation, ventilation, prone positioning, and re-intubation, or more likely a combination of these direct and indirect factors [16–18].

An elective tracheostomy can provide a closed respiratory circuit to facilitate weaning (when used with an inflated tube cuff), allowing for a more controlled wean than an attempt at primary extubation considered at high risk of failure. However, tracheostomy care still requires airway interventions that may be considered aerosol-generating and tracheostomy is not recommended in patients who are likely to require management in the prone position [19].

In addition to benefits for patients and staff, tracheostomy may also provide additional logistical and resource benefits for the hospital [20]. Patients with a tracheostomy typically require reduced or no sedation, reducing resource pressures on drugs, equipment and monitoring [21] and allowing for less intensive nursing care, as the patient may be able to assist in their own movement and self-care and be less dependent on multiple staff for re-positioning. During the pandemic, with increased demand for critical care beds compounded by staff absence through illness or shielding, there has been a reliance on non-critical care nurses and other healthcare professionals to assist within the ICU. Tracheostomy patients may be easier to care for than fully sedated patients, but adequate training must be undertaken to ensure these healthcare professionals are able to manage tracheostomies and identify any potential complications [22] and a role for nursing specialties already experienced with tracheostomies (head and neck surgery for example) may be beneficial.

It remains essential that the potential benefits of a tracheostomy are weighed against the potential burden for patients and risks to staff and local critical care resources. Tracheostomy should only be considered in patients recovering from critical illness who have a good chance of making a meaningful recovery.

## When to perform a tracheostomy?

Tracheostomies can pose a risk for the patient and the staff both in terms of insertion and subsequent management and, thus, the first priority when considering optimal timing for tracheostomy is whether the procedure will benefit the patient. Exposing the patient and staff to procedural risks when the patent is unlikely to survive does not benefit anybody. However, predicting which patients might benefit is difficult, both within and outside of the pandemic period. Considering that tracheostomy is indicated in those patients who have difficulty breathing and coughing independently, it is no surprise that mortality rates are high during critical illness and following ICU or hospital discharge [8]. Tracheostomies should only be undertaken in patients who are clinically improving. Patients requiring (or likely to require) prone positioning for respiratory failure should not be considered for tracheostomy due to the increased risk of tube displacement, occlusion, or impaired ability to identify tracheostomy-related complications in the prone position [23]. As with all complex decisions, a multidisciplinary approach is recommended [22].

**Fig. 1:**
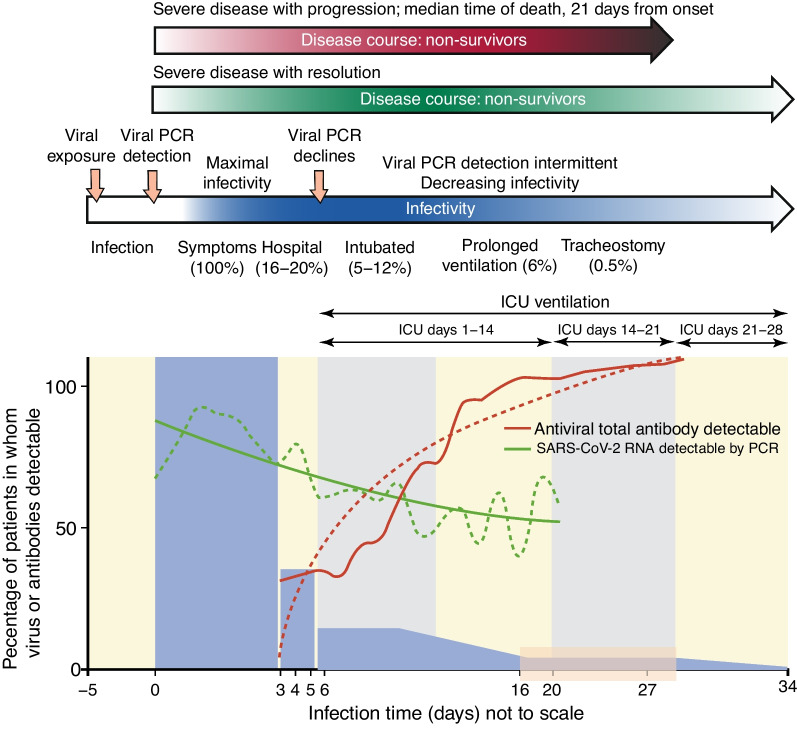
Typical clinical course, viral polymerase chain reaction (PCR), and antiviral antibody detection and infectivity of severe acute respiratory syndrome coronavirus 2 (SARS-CoV-2) infection. The transparent red box shows the suggested window for tracheostomy, on ICU days 10–21, which corresponds with 16–30 days from symptom onset. The solid bars and curves represent the proportion of all cases. Time zero is symptom onset (the x-axis is not to scale). Adapted from [19] with permission

**Fig. 2:**
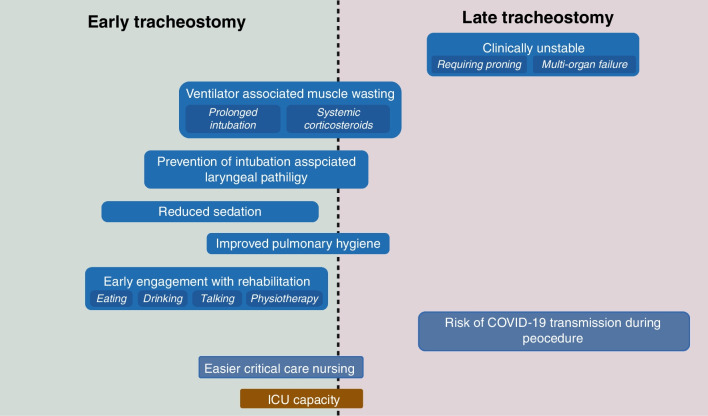
Factors favoring early or late tracheostomy in patients with COVID-19 disease. Patient factors (blue), staff factors (paler blue) and critical care resource factors (brown). Adapted from [19] (with permission)

Optimal timing for tracheostomy remains controversial in non-COVID-19 patients [24] and becomes more complicated in patients with COVID-19 due to the perceived risk of aerosol generation. Virological evidence suggests that the viral load falls from a peak associated with the onset of symptoms, although the window of detection is prolonged in critical illness [19] (Fig. 1). Considering that the insertion procedure is aerosol generating, therefore posing risks to operators and attending staff, delaying tracheostomy is likely to benefit staff by reducing the risk of transmission [25, 26]. This must be balanced against the potential benefits to the patient of early tracheostomy, such as reducing laryngeal injury and laryngeal dysfunction associated with prolonged tracheal intubation, reducing the cumulative burden of sedative agents, and promoting pulmonary hygiene through better secretion clearance [27–29]. Earlier tracheostomy also allows for outcomes that patients find particularly important, such as an earlier return to eating, drinking, talking, and engaging in proactive rehabilitation [30–32]. The factors favoring early or late tracheostomy in patients suffering from COVID-19 disease are summarized in Fig. 2.

**Fig. 3:**
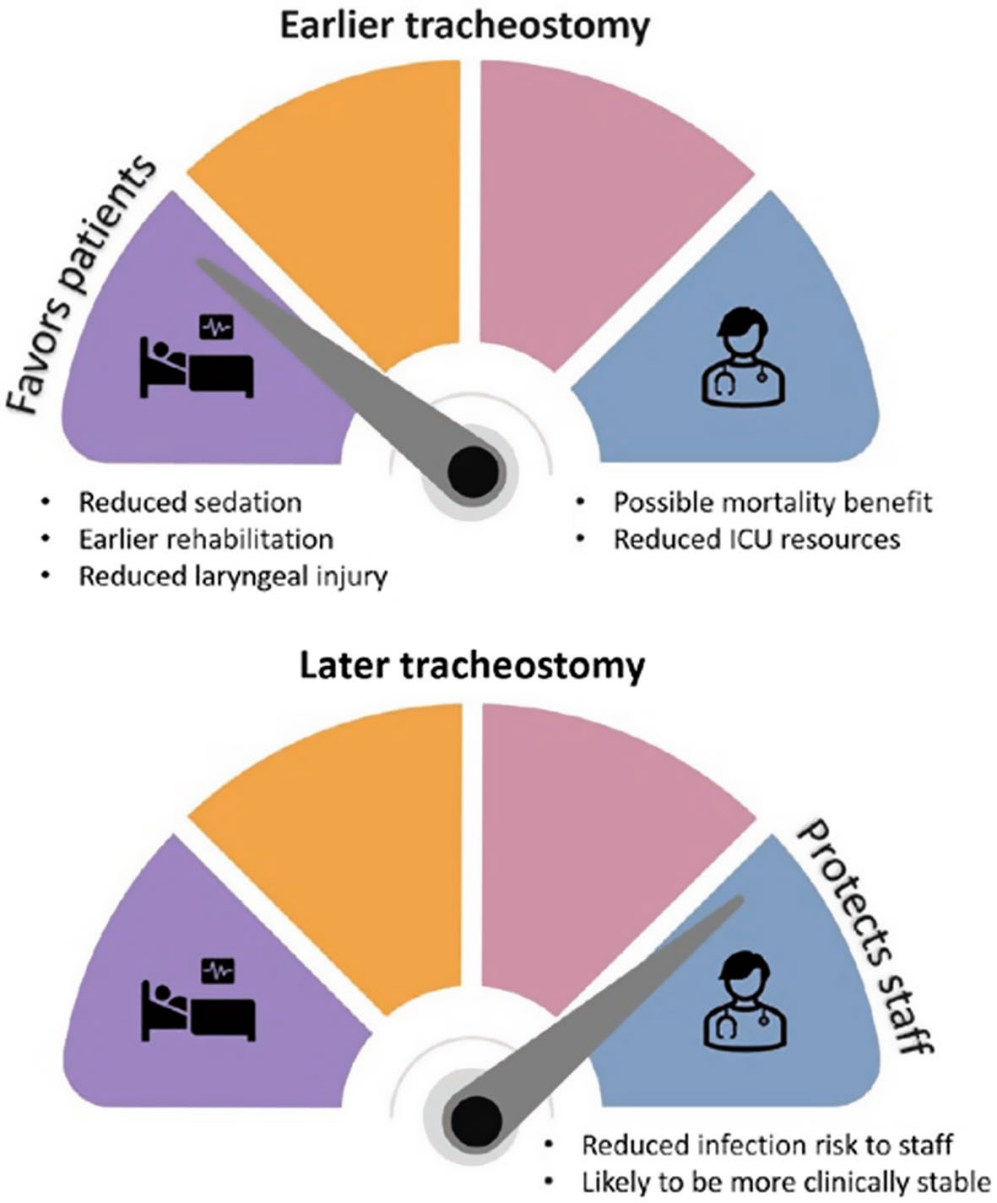
Balancing the benefit to the patient versus the risk to the staff

Early in the evolution of the pandemic, healthcare workers were rightly concerned about the risks of transmission during the tracheostomy insertion procedure, which has the potential to generate infectious aerosols. While many organizations in different countries advocated a cautious and therefore delayed approach to tracheostomy, a review of 26 international protocols demonstrated that timing for tracheostomy in COVID-19 varied from 3 to > 21 days [33]. The majority of protocols considered the implied infectivity of the critically ill patient, and as the predicted viral load and antibody response became more precisely characterized as the pandemic unfolded, most recommended a minimum of 14 days of mechanical ventilation prior to tracheostomy, balancing the risks of patient benefit with risks to staff (Fig. 3). As staff became more confident with personal protective equipment (PPE) and in managing patients with severe COVID-19, reports emerged indicating a role for early tracheostomy in some patients, with potential mortality benefits [5]. Although case selection for early tracheostomy will remain an evolving challenge, what is clear is that the timing of tracheostomy in the management of severe COVID-19-associated respiratory failure is returning to ‘business as usual’ [23].

The question of whether the patient is physiologically stable enough to tolerate the tracheostomy insertion is very relevant, as the time to discover that the patient will desaturate rapidly when ventilation is suspended is not when the neck has just been opened. Physiological deterioration can be anticipated to some degree in all tracheostomy procedures due to inadequate ventilation, transient suspension of ventilation and the lung de-recruitment associated with exchanging the trans-laryngeal tube for a new tracheostomy. However, the deranged respiratory physiology associated with severe COVID-19 may cause an exaggerated deterioration if the patient has not recovered sufficient physiological reserve to tolerate the procedure. An international expert panel suggested a pre-procedural ‘apnea test’ which attempts to simulate the procedural conditions and thus predict physiological readiness. Pre-oxygenation, followed by a trial of apnea in the ICU, with a FiO2 of 1. 0 and positive end-expiratory pressure (PEEP) of 5 cmH2O in the supine patient is suggested [19]. Rapid desaturation predicts a similar response during tracheostomy, indicating risk to the patient (and also to staff who may be required to undertake unplanned or additional airway interventions). Tracheostomy should be deferred in these circumstances. Importantly, the ability to conduct or tolerate an apnea trial should not replace multidisciplinary clinical judgement regarding the risks and benefits of undertaking tracheostomy in a given patient at a particular time [34].

## What is the best technique for inserting a tracheostomy?

The first consideration is location. Performing the procedure in the ICU minimizes patient movement, avoids the logistical considerations of assembling an operating room team, but brings technical obstacles such as the large ICU bed and deficiencies in trained assistance, the environment, and with equipment (Table 1). Ideally, aerosol-generating procedures in potentially infectious patients should occur in negative pressure isolation rooms. These are not universally available, but conditions are probably most closely replicated in the operating room suite [35].

**Table 1:** Advantages and disadvantages of performing a tracheostomy in the intensive care unit or operating room

Intensive care unit		Operating room	
Advantages	Disadvantages	Advantages	Disadvantages
No transfer	Positioning can be	Support available	Requires patient transfer
required	difficult	Controlled (aerosol)	(exposure risks to patient,
Timely (not	Less equipment	environment	staff and others)
dependent on	available for	Typically performed	Requires a surgeon to be
operating rooms)	complications	by surgeons (may save	available
Convenient for	Fewer resources in	ICU resources)	Takes an operating slot
ICU team	the event of a complication	Good lighting	
	Suboptimal lighting		
	Potential for distractions		

Second, the choice of insertion technique is essentially between an open surgical or a percutaneous approach. Hybrid approaches have been described and there are variations in all techniques described in the literature and facilitated by a wide range of equipment. An open technique was favored during the earlier severe acute respiratory syndrome (SARS), Middle East respiratory syndrome (MERS) and Avian influenza A (H5N1) viral pandemics based on low reported rates of transmission to operators [36, 37]. However, percutaneous techniques have progressed substantially in the last 20 years and many single centers have reported successful percutaneous approaches during the current pandemic, with apparently low rates of infectivity among attending staff [38–40].

The relative risks and benefits of percutaneous or surgical approaches have been debated in the literature for many years. Perceived benefits to the percutaneous technique are: familiarity to critical care staff; reduced air leak from the smaller stoma; fewer wound infections; and reduced bleeding complications.

However, percutaneous approaches involve: more airway manipulation than a surgical procedure; withdrawal of the tracheal tube risking extubation and aerosol generation; and, when combined with endoscopic visualization, may result in inadequate ventilation, significant upper airway gas leak and aerosol generation during the procedure. Perceived advantages to an open surgical technique relevant to COVID-19 are that it allows for a more controlled procedure, performed under direct vision. When combined with an expert anesthesiologist manipulating the tracheal tube in an ideal operating room environment, a surgical procedure may be safer.

Third, there may be situations where the patient’s condition favors a particular approach. Difficult neck anatomy, obesity or overlying thyroid gland or vessels are established indications for a surgical approach. What is less clear is how to manage patients who are receiving anticoagulants, receiving antiplatelet medication, or who are at an increased risk of bleeding—all of which are common dilemmas during the coronavirus pandemic. A percutaneous approach involves less dissection, a smaller stoma and thus a tamponading effect from the newly inserted tube, which may reduce post-procedural bleeding. A surgical approach offers more direct access to control specific bleeding sources, although the use of diathermy may be implicated in aerosolizing viral particles and diathermy should be kept to a minimum [19].

Fourth, modifications and considerations have been proposed to help reduce the risk of aerosol generation during tracheostomy insertion. Most advocate suspending ventilation at key steps in the insertion process: manipulation of the tracheal tube within the upper airway; opening the trachea; any dilatation of the stoma; and during insertion of the new tracheostomy tube [41]. This period of apnea, however brief, risks significant de-recruitment and hypoxia, and a period of pre-oxygenation can help mitigate this. During a surgical insertion, the tracheal tube with the balloon inflated may be advanced distally within the trachea beyond the tracheotomy, thus keeping the breathing circuit ‘closed’ [42]. Clear communication between all team members is essential. Communication may be impeded by PPE and planning, rehearsal, and simulated practice are recommended [19]. It is also recommended that the patient should be paralyzed, thus preventing coughing and unwanted movement and reducing peak airway pressures [19, 41].

Finally, the logistics of managing multiple critically ill patients in our hospitals may influence the choice of technique simply through the availability of trained staff to undertake the procedure, with many centers reporting a significant rise in the number of surgical procedures undertaken during the pandemic [23, 39–42].

Because of the aerosol-generating nature of this procedure, it is imperative that appropriate PPE is always worn by whoever undertakes the tracheostomy insertion, and only essential staff are present in the immediate environment [36]. What is clear is that more research is needed to understand the optimal technique for a particular set of circumstances and while we await clearer answers, practitioners are advised to do what works best in their institution, with their local resources, practice and expertise used optimally following multidisciplinary discussion between all stakeholders.

## Subsequent management of a patient with a tracheostomy

For patients with COVID-19 who have a tracheostomy, the aims of care are to minimize airway interventions and potential aerosol generation, whilst maintaining standards of safe care and ensuring that patients are proactively rehabilitated. All interventions should involve thorough planning to reduce risks to both patients and staff, and care should be performed by staff experienced with tracheostomy care [22]. Strategies to minimize aerosol production have been proposed, which include reducing ‘routine’ suction and inner cannula care to a minimum [43], using ‘closed suction’ systems, and using heat and moisture exchange (HME) filters in ventilator circuits instead of heated water-based ‘active’ humidification systems [44]. All of these strategies require regular review for each patient. If, for example, secretions become thicker, additional therapies such as mucolytic drugs, nebulizers, or switching to active humidification may be required [19].

It is recognized that tracheostomy weaning in a patient with COVID-19 provides a unique challenge. In non-COVID-19 patients, the process of weaning would involve gradually decreasing the ventilatory support alongside periods of cuff deflation, strategies which clearly promote aerosol generation [43, 45]. A ‘cuff-up’ strategy is initially suggested for patients with COVID-19 disease, and only when the patient is deemed at lower risk of infectivity should the cuff be deflated [18]. Others have argued that this cautious approach disadvantages patients and may slow their recovery and laryngeal rehabilitation, instead advocating for adequate staff PPE in dedicated clinical areas alongside face and tracheostomy shields to reduce aerosol risks [19]. The optimal strategy is yet to be determined, but will be heavily influenced by local infrastructure, the environment and the experience and confidence of attending staff.

**Fig. 4:**
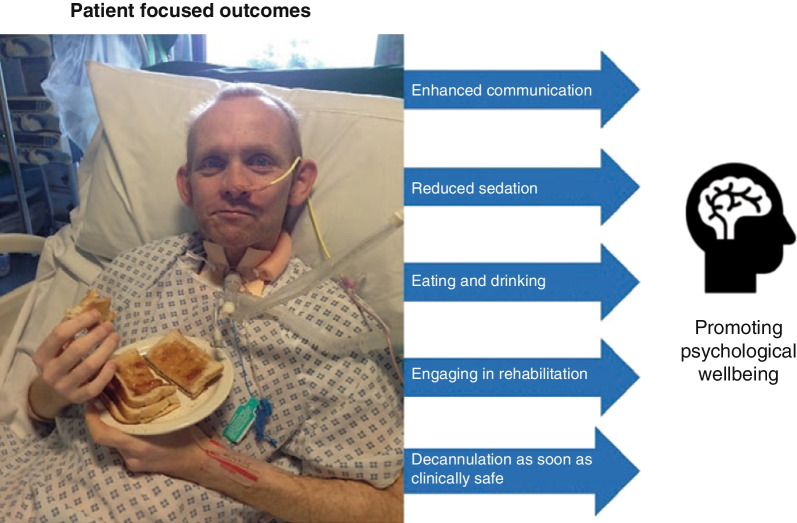
Patient-focused outcomes in tracheostomy care (the patient provided permission to publish the photo)

The deep psychological impact of an ICU stay on patient wellbeing during and beyond the ICU is well documented [46]. However, COVID-19 has posed new challenges by limiting the interactions that patients can have with family and staff. Speech for patients with a tracheostomy usually requires cuff deflation, risking aerosolization if positive pressure ventilation or ventilatory support is still required. Innovative communication methods include communication boards, ‘speaking’ tracheostomy tubes, above-cuff vocalization strategies, the use of an electrolarynx and other alternative communication devices [47]. Oral feeding, following a rigorous swallow assessment may also provide psychological benefit [32]. Such patient-focused outcomes are highlighted in Fig. 4.

Safe decannulation should occur as soon as clinically possible [19]. Some have argued that decannulation should occur only following negative COVID-19 test results [48], but this may not be feasible if intensive care beds are limited, especially as complete viral clearance may take a significant period of time [49], thus delaying necessary patient care.

## Conclusion

Tracheostomy is an important therapeutic intervention in the critically ill. The coronavirus pandemic has seen a significant increase both in the proportion of critically ill patients who become tracheostomy candidates and the absolute numbers of patients undergoing tracheostomy. Decisions surrounding candidacy, optimal timing, optimal technique and the optimal multidisciplinary aftercare of tracheostomies in the critically ill can be complex outside of the pandemic—a situation made yet more complex by the potential to transmit disease by infectious aerosols from those with COVID-19. After a steep learning curve, our multidisciplinary community is well placed to protect healthcare staff while ensuring that the best possible, pro-active care is delivered to the many patients who will benefit from tracheostomy as part of their critical illness management. Many questions remain, and continued tracheostomy research, global collaboration and quality improvement is imperative [50] to ensure the boundaries of quality tracheostomy care continue to be pushed for the benefit of our patients.

## Data Availability

Any specific data belonging to the authors discussed in this article is available on request.
